# Strain Comparison in Rats Differentiates Strain-Specific from More General Correlates of Noise-Induced Hearing Loss and Tinnitus

**DOI:** 10.1007/s10162-021-00822-2

**Published:** 2021-11-18

**Authors:** L. Koch, B. H. Gaese, Manuela Nowotny

**Affiliations:** 1 Institute of Cell Biology and Neuroscience, Goethe University, Frankfurt am Main, Germany; 2 Animal Physiology Group, Institute of Zoology and Evolutionary Research, Friedrich-Schiller-University, Jena, Germany

**Keywords:** Strain comparison, ABR, Noise exposure, Acoustic startle response, Gap-PPI, Tinnitus

## Abstract

Experiments in rodent animal models help to reveal the characteristics and underlying mechanisms of pathologies related to hearing loss such as tinnitus or hyperacusis. However, a reliable understanding is still lacking. Here, four different rat strains (Sprague Dawley, Wistar, Long Evans, and Lister Hooded) underwent comparative analysis of electrophysiological (auditory brainstem responses, ABRs) and behavioral measures after noise trauma induction to differentiate between strain-dependent trauma effects and more consistent changes across strains, such as frequency dependence or systematic temporal changes. Several hearing- and trauma-related characteristics were clearly strain-dependent. Lister Hooded rats had especially high hearing thresholds and were unable to detect a silent gap in continuous background noise but displayed the highest startle amplitudes. After noise exposure, ABR thresholds revealed a strain-dependent pattern of recovery. ABR waveforms varied in detail among rat strains, and the difference was most prominent at later peaks arising approximately 3.7 ms after stimulus onset. However, changes in ABR waveforms after trauma were small compared to consistent strain-dependent differences between individual waveform components. At the behavioral level, startle-based gap-prepulse inhibition (gap-PPI) was used to evaluate the occurrence and characteristics of tinnitus after noise exposure. A loss of gap-PPI was found in 33% of Wistar, 50% of Sprague Dawley, and 75% of Long Evans rats. Across strains, the most consistent characteristic was a frequency-specific pattern of the loss of gap-PPI, with the highest rates at approximately one octave above trauma. An additional range exhibiting loss of gap-PPI directly below trauma frequency was revealed in Sprague Dawley and Long Evans rats. Further research should focus on these frequency ranges when investigating the underlying mechanisms of tinnitus induction.

## INTRODUCTION

In addition to increasing age, the strongest determinant for hearing loss is damage to the sensory cells within the cochlea caused by noise exposure (Liberman and Dodds 1984). A malfunction of these peripheral structures leads to decreased transmission of auditory information to higher processing centers, thereby impairing hearing ability and leading to perceptual anomalies such as tinnitus, hyperacusis, or hidden hearing loss (Sheppard et al. 2020).

In the adult human population, tinnitus prevalence ranges between 3 and 15% (Adams et al. 1999; Heller 2003; Hoffman and Reed 2004; Shargorodsky et al. 2010), with 0.5–2.8% experiencing a severe, debilitating form of tinnitus (Baguley et al. 2013). Noise-induced hearing loss often accompanies tinnitus (Eggermont and Roberts 2015). To date, no reliable, effective therapy is available for tinnitus patients (Sheppard et al. 2020), partially because the underlying pathological changes of tinnitus induction and persistence remain unclear.

Almost all experimental approaches investigating tinnitus mechanisms involve rodent animal models. Among them are mice (Hickox and Liberman 2014; Liberman and Liberman 2015; Nowotny et al. 2017; Park et al. 2020), gerbils (Nowotny et al. 2011; Kiefer et al. 2015; Schilling et al. 2017; Jeschke et al. 2021), hamsters (Chen et al. 2013; Manzoor et al. 2013), guinea pigs (Mulders et al. 2014; Hockley et al. 2020), and rats (Turner et al. 2006; Caspary et al. 2008; Lobarinas et al. 2013; Möhrle et al. 2019; van Zwieten et al. 2021).

After noise exposure, several different types of physiological changes can occur along the auditory pathway. The demyelination or loss of afferent fibers from the inner ear is reflected in a reduced auditory brainstem response (ABR) wave I amplitude (Schaette and McAlpine 2011; Wan and Corfas 2017). Due to reduced cochlear output, noise-induced cochlear damage (mainly to inner hair cells) can lead to increased spontaneous activity in subcortical structures and synchronized gain in the auditory cortex (Bauer 2004; Roberts et al. 2010; Eggermont 2016). Peripheral changes build up in the brain and may ultimately lead to permanent changes that have often been interpreted as chronic tinnitus (Kaltenbach 2011).

The heterogeneity of results in terms of affected frequencies and the amount of damage caused by noise trauma described thus far have prevented the emergence of distinct physiological, cellular, or molecular markers for tinnitus (Brozoski and Bauer 2016). A clear indication of tinnitus is expected at the behavioral level. Differential tests were applied that are mostly based on investigating an altered concept of silence when tinnitus is present: operant conditioning (e.g., Jastreboff et al. 1988) or reflex-based methods such as the Preyer reflex (Berger et al. 2013; Hockley et al. 2020) and gap-prepulse inhibition of the acoustic startle response (gap-PPI, e.g., Turner et al. 2006). Gap-PPI is based on the assumption that the startle-inhibiting effect of a gap-prepulse is impaired after trauma in frequency ranges where a presumed tinnitus perceptually fills the silent gap. Other studies, however, found characteristics of (at least co-occurring) hyperacusis (Chen et al. 2013; Salloum et al. 2016). Still, it is also important to control for the interfering possibility of a permanent hearing loss.

While several of these studies found clear behavioral effects after trauma, only a percentage of animals (between 33 and 86%) usually develop behavioral indications of hearing anomalies such as tinnitus (e.g., Rüttiger et al. 2013; Pace et al. 2016). The frequency dependence of induced tinnitus sensation is very heterogeneous between studies, only partially resulting from differences in the frequency ranges used for trauma induction. In previous studies, a similar spectral composition of noise exposure (centered at 16 kHz) led to different tinnitus frequencies in rats (10, 20 and 32 kHz, respectively, Brozoski and Bauer 2005; Turner et al. 2006; Zheng et al. 2014; Lobarinas et al. 2015).

Considering the development of transgenic rat models that can be used for optogenetic research (Azzopardi et al. 2018; Keppeler et al. 2020), it is of utmost importance to characterize prevalent variations between strains to identify the most suitable animal model. Many studies have investigated the ABRs in different rat strains under untreated conditions (Borg 1982; Chen and Chen 1990; Overbeck and Church 1992). To date, no comparative study has investigated the effects of noise exposure on ABR thresholds and waveforms in different rat strains. The same applies for the measurement of the acoustic startle response (ASR). Strain comparisons have been performed (Varty and Higgins 1994; Acri et al. 1995; Faraday et al. 1999; Palmer et al. 2000; Rybalko et al. 2012), but not in combination with noise trauma.

By comparing different rat strains, the focus of the present study was to identify reliable markers and parameters for noise-induced hearing loss and loss of gap-PPI to characterize tinnitus sensation. Therefore, using electrophysiological and behavioral methods, we studied the differential effects of noise exposure on the auditory systems of four outbred rat strains: Sprague Dawley, Wistar, Lister Hooded, and Long Evans. We performed ABR recordings to determine hearing thresholds before and directly after noise exposure to ensure a clear trauma induction in the desired frequency range, and finally monitored threshold recovery over several weeks. ABR waveform components and amplitudes were examined to find strain-dependent differences. Frequency-specific gap-PPI was compared between before and after trauma and evaluated for a post-trauma reduction of inhibition as a possible behavioral correlate of tinnitus.

## MATERIAL AND METHODS

### Animals

Four different outbred rat strains of the species *Rattus norvegicus* were investigated in this study using female animals at approximately 8 weeks of age at the beginning of the experiments. Based on previous investigations of ASR, it seemed not necessary to test the animals’ estrous stage (Adams et al. 2008). Rats from the Sprague Dawley (SD, *n* = 8), Wistar (WI, *n* = 9), Lister Hooded (LH, *n* = 8), and Long Evans (LE, *n* = 8) strains were purchased from Charles River Laboratories (Sulzfeld, Germany). One animal from the Lister Hooded group died before the last ABR measurement. However, ASR measurements were performed in all animals. Rats were housed in a specific pathogen-free facility in pairs or triplets, divided by strain and maintained on an inverted 12-h light/dark cycle (dark phase starting at 7 a.m.) in a temperature- and humidity-controlled room. Experiments took place during the dark phase. Otoscopic examination was performed before experimental manipulations to assess the condition of the middle ear. Food and water were provided *ad libitum*. All procedures were in accordance with ethical standards for the care and use of animals in research, the German Animal Welfare Act, and were approved by the Regierungspraesidium Darmstadt (FU/Anz. 1002).

### Auditory Brainstem Response

For the measurement of auditory brainstem responses, animals were anesthetized with a combination of 92 mg/kg body weight (BW) ketamine and 3.7 mg/kg BW xylazine administered intraperitoneally. The depth of anesthesia was evaluated with the toe-pinch reflex and by checking for vibrissae movements. A state of light anesthesia was maintained with continuous intraperitoneal infusion of the same anesthetic solution at a rate of 60 µl/h and approximately 80 µl/h for Lister Hooded rats during the experiments (syringe pump AL1000-220, World Precision Instruments), while the animals were placed on a heating pad inside a soundproof chamber.

ABRs were determined using pure-tone stimuli at frequencies of 6, 12, 16, 20, 26, and 32 kHz (10 ms duration, r/f time: 0.5 ms) and a click stimulus (0.5 ms duration, r/f time: 0.05 ms). Measurements were taken immediately before and directly after trauma induction and 1, 4, and 8 weeks after noise exposure. Acoustic stimuli were generated by a sound card (Juli@ 24-bit, 192 kHz, ESI Audiotechnik GmbH), amplified (RB-970BX, Rotel) and presented via a loudspeaker (R2904/70000, Scan-Speak A/S) placed 10 cm lateral to the left ear of the anesthetized animal. Silver wire electrodes were used to pick up ABRs at two different subcutaneous positions: (i) behind the left bulla and (ii) above the contralateral inferior colliculus. A ground electrode was placed near the root of the tail. The inverted signal of the electrode placed at the inferior colliculus was added to the signal of the electrode at the bulla. Measurements for each stimulus were averaged across 400 repetitions (inter-stimulus interval 100 ms), and all signals were amplified and bandpass filtered (0.3–3 kHz, EX-1 Differential Amplifier, Dagan Corporation). The signal was fed back into the sound card, sampled at a rate of 96 kHz, and analyzed in software written in MATLAB (The Mathworks Inc.). ABR-based hearing thresholds were detected by visual inspection at the lowest intensity at which a pronounced response was present.

### Noise Exposure

A centrally placed horn speaker (HTH 8.7-8 Ω, Visaton GmbH & Co. KG) approximately 10 cm above the head of the anesthetized rat was used to induce binaural free-field acoustic trauma. The narrowband overstimulation was centered at 16 kHz with a frequency bandwidth of 0.5 oct and presented at an intensity of 115 dB SPL (peak-to-peak) for one hour.

### Acoustic Startle Response Measurements

Measurements based on the acoustic startle reflex were used for behavioral determination of a possible tinnitus percept. ASR amplitudes were compared between pre-trauma and 7 weeks after noise exposure. Rats were placed inside a custom-built wire mesh cage (9 × 7 × 18 cm) mounted on a strain gage platform (Platform W/load Cell PHM-250, MED Associates Inc.) inside a sound-attenuating booth (ENV-1080MD, MED Associates Inc.). Software written in MATLAB generated the acoustic stimuli and recorded motion responses from the animals. Sound stimuli were delivered by a sound card (sampling rate 96 kHz, Fireface 400, RME Audio AG), amplified (RMB 1506, Rotel) and broadcasted via a loudspeaker (MHT 12-8 Ω Ribbon Tweeter, Visaton GmbH & Co. KG) placed 10 cm above the animal’s head. The motion signal elicited by the startle response was transduced, pre-amplified (Transducer and Amp PHM-250B, MED Associates Inc.), amplified (*g* =100, custom-built), and eventually fed back into the sound card.

Several different behavioral paradigms were used to assess hearing characteristics based on the startle response. The startle stimulus in all paradigms was a broadband noise pulse (BBN: 2–20 kHz) with a duration of 20 ms (r/f time 0.0001 ms). First, we used an input/output (I/O) function of the startle response at intensities ranging from 65 to 115 dB SPL in 10 dB steps to test the startle threshold and amplitudes. The presentation was in random order, with each stimulus presented 20 times. This was followed by measuring gap-prepulse inhibition (gap-PPI) of the startle response with narrowband background noise (± 0.25 oct) at 80 dB SPL. Aforementioned intensity was chosen to ensure salience of the background stimulation despite the occurrence of hearing loss after noise trauma (Hayes et al 2014). The same center frequencies as used for ABR measurement (6, 12, 16, 20, 26, 32 kHz and BBN) were used for this paradigm. Gap length was set to 500 ms, and gaps ended 50 ms before the startle pulse, based on previous findings (Steube et al. 2016). For each frequency, trials with and without gaps were examined randomly to assess the percentage gap-PPI (see Eq. 1). Each frequency and gap configuration was tested 16 times (random order). The whole paradigm was split into two sessions and measured on consecutive days.

1 $$\frac{\left(\mathrm{ASR\;without ~gap}\right)-\left(\mathrm{ASR\;with ~gap}\right)}{\mathrm{ASR\;without ~gap }\times 100}=\mathrm{inhibition }[\mathrm{\%}]$$

All startle sessions were performed during the morning hours (7 a.m. to 12 p.m.) to minimize the influence of circadian fluctuation on startle behavior (Chabot and Taylor 1992; Basinou et al. 2017). Startle responses were adapted with eight stimulus presentations before the actual measurement began (Pilz and Schnitzler 1996; Koch 1999; Gaese et al. 2009) to minimize the effects of short-term habituation during measurements. Adaptation trials were excluded from the analysis.

### Statistical Analysis

All statistical analyses were performed using JMP 7.0 (SAS Institute Inc.). All tested datasets met ANOVA requirements, including testing for normal distribution (Shapiro–Wilk test).

The general strain comparison before noise trauma was performed by running a two-way ANOVA testing for influences of strain and frequency followed by *post hoc* testing using independent contrasts with Bonferroni-Holms correction for multiple comparisons. Data before and after acoustic overstimulation were analyzed with three-way ANOVAs (including the factor time), again followed by *post hoc* analysis. No statistical analysis was performed for the peak analysis because the sample size was too small. Levels of significance are indicated as follows: *p* < 0.05 = *, *p* < 0.01 = **, *p* < 0.001 = ***. All averaged data are noted as the mean ± standard deviation, unless otherwise stated.

## RESULTS

In the present study, we describe hearing characteristics before and after acoustic overstimulation in four different rat strains. ABR was determined before and directly after trauma and then 1, 4, and 8 weeks after trauma to identify electrophysiological changes over time, while ASR was measured before and seven weeks after noise exposure to study behavioral changes and the development of tinnitus.

### Basic Hearing Characteristics Differ Among Rat Strains

**Fig. 1:**
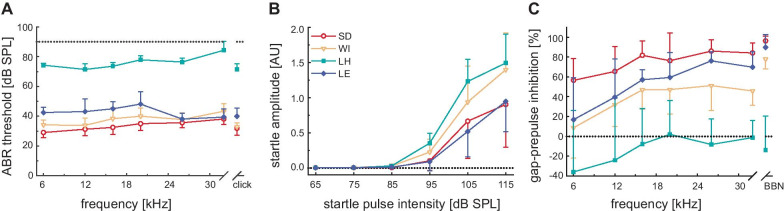
Strain-dependent differences in basic hearing characteristics. **A** Average ABR hearing thresholds of four different rat strains: Sprague Dawley (SD, red open circles, *n* = 8), Wistar (WI, orange open triangles, *n* = 9), Lister Hooded (LH, cyan squares, *n* = 7 in 1A, *n* = 8 in 1B and 1C), and Long Evans (LE, blue diamonds, *n* = 8). ABR test tones had intensities up to 90 dB SPL (dotted horizontal line). **B** Input/output function of the acoustic startle response. Average peak-to-peak amplitudes of the startle responses are shown as functions of the stimulus level. The dotted horizontal line indicates a startle amplitude of 0. **C** Frequency-specific gap-prepulse inhibition of the startle response measured at six different frequencies and BBN (broadband noise, 2–20 kHz). The dotted horizontal line indicates no inhibition (i.e., gap-PPI of 0%). Details of significant differences among rat strains occurring in data are mentioned in the main text

ABR hearing thresholds and acoustic startle responses were determined before noise exposure to provide a baseline comparison of the four different rat strains. We found significant effects of strain and frequency on ABR hearing thresholds (two-way ANOVA: strain: *F*_3,224_ = 923.11, *p* < 0.001, frequency: *F*_7,224_ = 9.61, *p* < 0.001). Furthermore, the interaction between strain and frequency was statistically significant (two-way ANOVA: *F*_21,224_ = 2.54, *p* < 0.001). The albino strains Sprague Dawley (*p* < 0.001) and Wistar (*p* < 0.05) had significantly lower ABR thresholds in the frequency range below 26 kHz than the pigmented strains Long Evans and Lister Hooded (Fig. 1A). Especially the threshold in Lister Hooded rats was much higher (at approximately 75 dB SPL) and significantly different from those of all other rat strains (*p* < 0.001).

Investigating the I/O-function of the acoustic startle revealed that the threshold to elicit a startle response was 95 dB SPL for all strains (Fig. 1B). However, startle amplitudes showed a strong strain dependency (two-way ANOVA: *F*_3,168_ = 9.84, *p* < 0.001). At a stimulus intensity of 115 dB SPL, Lister Hooded rats had the highest startle amplitude of approximately 1.5, while the lowest amplitude (approximately one-third lower) was elicited in Sprague Dawley rats. Startle amplitudes in Lister Hooded (*p* < 0.001) and Wistar rats (*p* < 0.01) at this stimulus intensity were significantly higher than those in Sprague Dawley and Long Evans rats. A similar pattern was observed at 105 dB SPL, where Lister Hooded rats had significantly higher (*p* < 0.001) amplitudes than Long Evans and Sprague Dawley rats. Despite their insensitive hearing, as measured by the ABR threshold, Lister Hooded rats had the same startle threshold as the other strains and even showed the strongest reactivity to high-SPL startle stimuli.

In an additional startle paradigm, gap-prepulse inhibition, we found that strain and frequency had significant effects (two-way ANOVA: strain: *F*_3,224_ = 67.54, *p* < 0.001, frequency: *F*_7,224_ = 9.44, *p* < 0.001). Animals of the Sprague Dawley strain exhibited the highest levels of inhibition, indicating a robust gap detection ability (mean inhibition: 78.4%; Fig. 1C). Long Evans and Wistar rats had slightly lower levels of inhibition (mean inhibition LE: 58.4%, WI: 44.2%), while Lister Hooded rats showed no inhibition, with rates of approximately 0%, which was significantly (*p* < 0.05) different from all other rat strains.

Taken together, the data from the evaluation of basic hearing characteristics indicated a complex strain-dependent pattern: the ABR threshold was not predictive of the ability to elicit a startle response. Furthermore, startle amplitude was not predictive of the amount of gap-induced inhibition, as most prominently seen in Lister Hooded rats. Despite their elevated hearing thresholds, they responded with the strongest startle amplitudes; however, they were unable to detect a silent gap in continuous background noise.

**Fig. 2:**
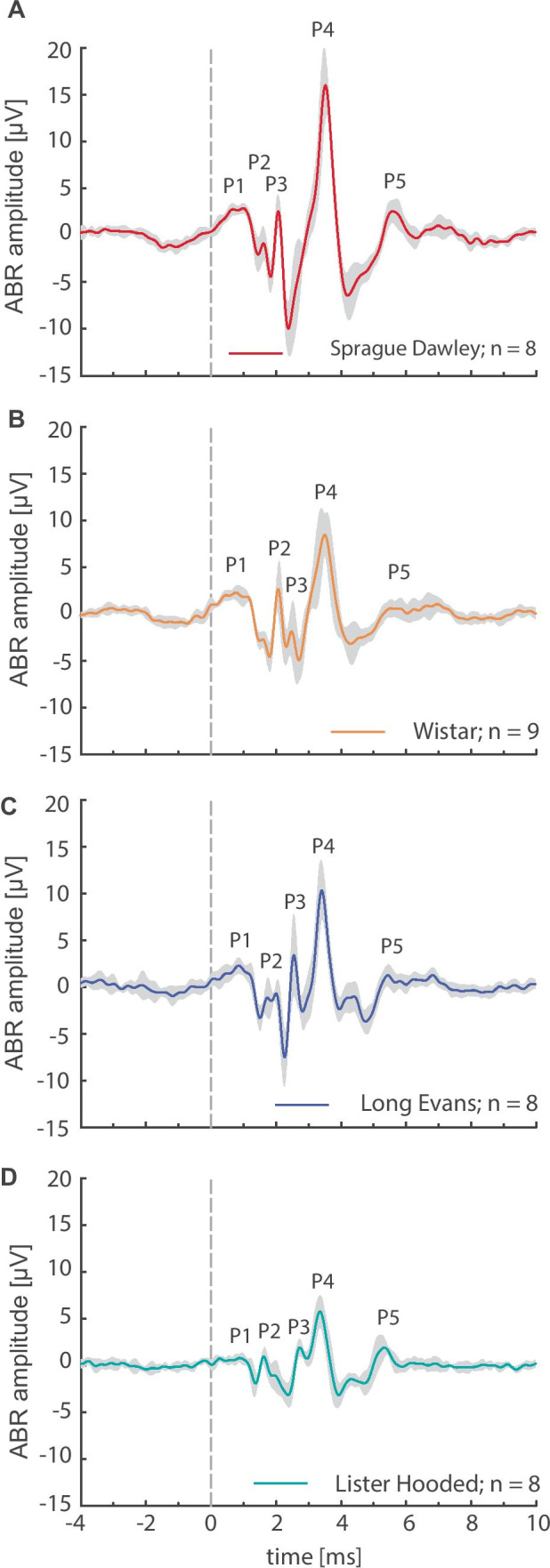
Strain-dependent differences in ABR waveforms. ABR waveforms elicited by a click stimulus (400 repetitions, standard deviation indicated as gray shadow) are presented at 80 dB SPL. Waveforms are shown for Sprague Dawley (**A**), Wistar (**B**), Long Evans (**C**), and Lister Hooded rats at pre-trauma values (**D**). The vertical dashed lines indicate stimulus onset

ABR waveforms at suprathreshold level are complex, including components of different latency that can be related to processing at different levels along the ascending auditory pathway. These were investigated for strain-dependent differences regarding their amplitudes. ABR waveforms elicited by pure tone and click stimuli were characterized by a sequence of peaks and troughs. For all strains, the detailed analysis revealed a click-induced ABR waveform with five vertex-negative peaks (P1–P5; Fig. 2). The different peaks represent cochlear input as auditory nerve activity (P1), neural responses from the cochlear nucleus (P2), superior olivary complex (P3), lateral lemniscus and inferior colliculus (P4), and medial geniculate body (P5, Henry, 1979; Overbeck and Church, 1992). However, this general waveform elicited by an 80 dB SPL click was characterized by clear differences in the response amplitude between the different rat strains. This was especially obvious for the highest peak (P4) found approximately 3.7 ms after stimulus onset. The maximum amplitude of P4 was strain-dependent (ANOVA: *F*_3,29_ = 16.73, *p* < 0.001) and significantly higher in Sprague Dawley rats (23.3 ± 5.8 µV, *n* = 8, *p* < 0.001, *post hoc*; Fig. 2A) than in all other strains. Wistar (14.3 ± 3.7 µV, *n* = 9; Fig. 2B) and Long Evans rats (14.1 ± 3.5 µV, *n* = 8; Fig. 2C) had almost comparable maximum P4 amplitudes, while the smallest peak amplitude was observed in Lister Hooded rats (9.6 ± 2.0 µV, *n* = 8; Fig. 2D). These peak-to-peak values for the mean peak amplitudes were determined for each animal at their individual timing, while the waveforms in Fig. 2 show the mean values for each rat strain. Therefore, individual maximum values are higher than the averaged wave values. Clear differences in the occurrence of the different wave peaks were found for the peak amplitude of P3, which was clearly detectable in all strains except for Sprague Dawley rats. Note that the form of the standard deviation (depicted as a gray shadow around waveforms in Fig. 2) clearly indicates that P3 must have occurred in some individual animals of the Sprague Dawley strain (Fig. 2A). Further analysis revealed that this peak became more distinct with increasing stimulus frequency above 80 dB SPL. In summary, analysis of ABR waveforms at suprathreshold level revealed distinct strain differences especially in the P4 peak component, with P4 amplitude being notably high in Sprague Dawley rats.

Taken together, evaluation of basic hearing characteristics revealed an interesting pattern with lowest ABR thresholds in Sprague Dawley rats that were linked to higher gap-PPI values (Fig. 1A, C) and largest ABR amplitudes (Fig. 2A). On the other hand, most elevated ABR thresholds in Lister Hooded rats were associated with lowest gap-PPI (Fig. 1A, C) and smallest ABR amplitudes (Fig. 2D).

### Trauma-Induced Changes in ABR and Startle Response

**Fig. 3:**
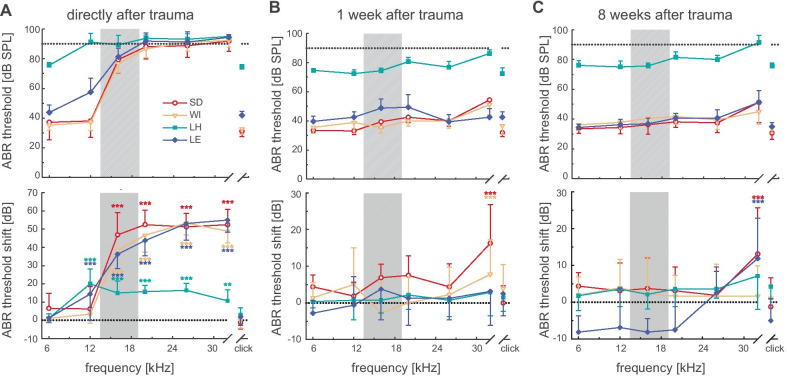
ABR threshold and threshold shift after acoustic overstimulation. The gray bar indicates the frequency range of the noise band used for acoustic overstimulation (noise: 16 kHz ± 0.25 oct for 1 h at 115 dB SPL). Depicted are average ABR thresholds (upper row) and threshold shifts (lower row) as functions of the test frequency **A** directly after trauma, **B** one week after trauma, and **C** 8 weeks after trauma. Note different scaling of the *y*-axis in the lower row in **A** compared to **B** and **C**. SD = Sprague Dawley, red open circles, *n* = 8, WI = Wistar, orange open triangles, *n* = 9; LH = Lister Hooded, cyan squares, *n* = 7; LE = Long Evans, blue diamonds, *n* = 8

For investigating trauma-induced changes, ABR hearing thresholds were reassessed within the first hour after the induction of noise trauma and then 1, 4, and 8 weeks after noise exposure (Fig. 3). In a three-way ANOVA, we found significant effects of the factors strain (*F*_3,1080_ = 3694.02, *p* < 0.001), time (*F*_4,1080_ = 1274.33, *p* < 0.001), and frequency (*F*_7,1080_ = 296.40, *p* < 0.001) on hearing thresholds after noise exposure. We observed significantly elevated ABR thresholds directly after trauma in all strains (Fig. 3A). However, the lowest significantly affected stimulus frequencies depended on rat strain and were 12 kHz in pigmented animals (Lister Hooded and Long Evans) and 16 kHz in albino animals (Sprague Dawley and Wistar).

One week after trauma, ABR thresholds in pigmented strains were no longer significantly different from pre-trauma values at all frequencies tested. However, albino strains still exhibited a significant threshold elevation at 32 kHz (Fig. 3B). Three weeks later (data not shown), no more significant threshold shifts compared to pre-trauma thresholds were found in the lower frequency range in all four strains. Yet, the threshold elevations observed for Sprague Dawley and Wistar rats persisted. At 32 kHz, they still demonstrated, together with Long Evans rats, a significant threshold elevation of 9 ± 9 dB (*n* = 25).

ABR thresholds 8 weeks after trauma revealed a persistence of the previously observed significant threshold elevation at 32 kHz in Sprague Dawley and Long Evans rats (Fig. 3C). Conversely, the hearing thresholds in the Wistar strain were no longer significantly different from pre-trauma values. Long Evans rats showed more sensitive hearing thresholds in the frequency range below 26 kHz (Fig. 3C). In summary, ABR thresholds directly after trauma were significantly elevated at frequencies at and above 12 kHz in pigmented and at and above 16 kHz in albinotic strains. Hearing thresholds recovered within 1 week except for 32 kHz in the albino strains. Eight weeks after noise exposure, hearing thresholds remained elevated at 32 kHz only in Sprague Dawley and Long Evans rats.

**Fig. 4:**
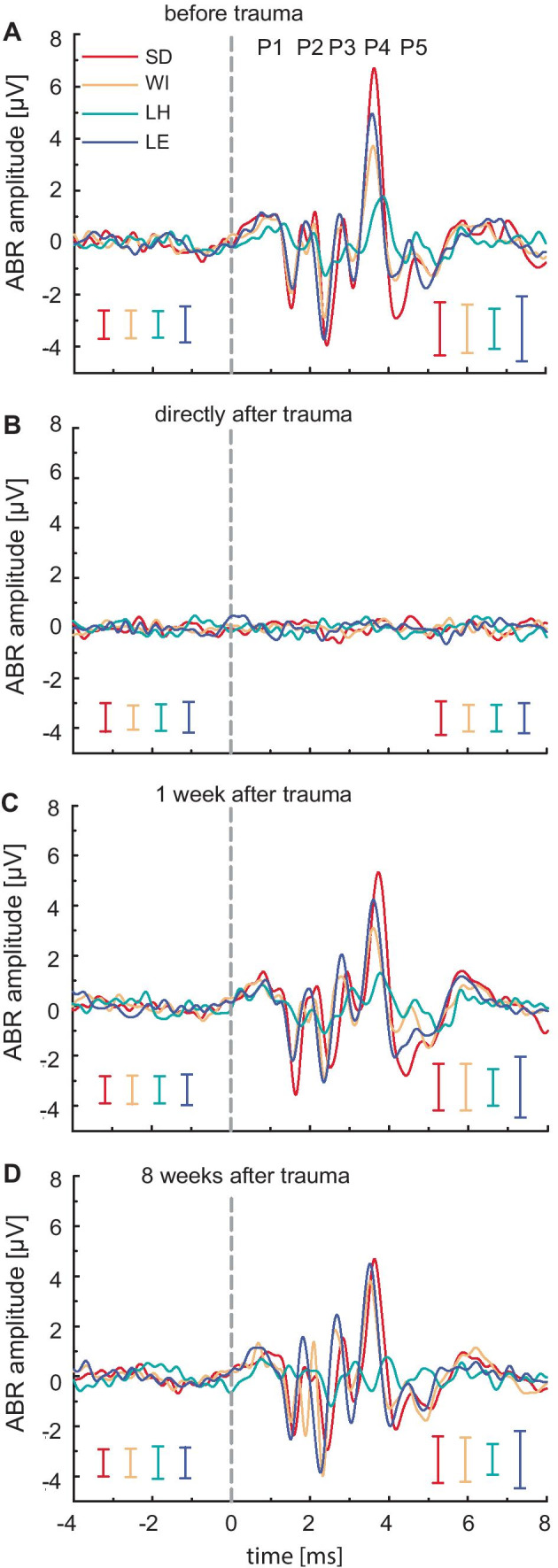
Comparison of ABR waveforms at different time points at 26 kHz and 80 dB SPL. **A** Before trauma, **B** directly after trauma, **C** 1 week after trauma, and **D** 8 weeks after trauma. SD = Sprague Dawley, *n* = 8; WI = Wistar, *n* = 9; LH = Lister Hooded, *n* = 7; LE = Long Evans, *n* = 8. Prominent peaks are displayed in **A**. Vertical dashed lines indicate stimulus onset, and colored bars show the standard deviation in a control window 7 to 1 ms before stimulus onset (left group in each plot) and during the response (right group, 0 to 6 ms after stimulus onset)

To identify trauma-induced changes in ABR waveforms apart from threshold shifts, we analyzed the waveforms elicited with 80 dB SPL stimulation at 26 kHz in detail. This frequency was chosen because (i) this stimulus frequency is well above the noise trauma band (13.4–19.0 kHz) and (ii) only a temporary threshold shift was found at 26 kHz. While there was a clear ABR waveform in the measurements before trauma (Fig. 4A), directly after noise exposure, no ABR response could be discerned from the noise level for any of the tested strains (Fig. 4B). The recovery of ABR amplitudes in Lister Hooded rats was weaker than in the other strains, and high ABR thresholds prevented further peak analysis. Thus, data from Lister Hooded rats are plotted, but the focus will be on the three remaining strains: Sprague Dawley, Wistar and Long Evans. One week after trauma, overall responses reappeared but were lower in response amplitude, and a reduction in response latency compared to pre-trauma values was detectable (approximately − 0.3 ms; Fig. 4C). Eight weeks after trauma (8 wat), the amplitudes of P4 became more similar among strains, and the initial strain-dependent variability was weaker. In contrast, P3 amplitudes exhibited higher variability among strains in terms of amplitude and latency (Fig. 4D). After acoustic trauma, decreased mean latencies for P3 and P4 were found in Wistar (8 wat P3: − 0.09 ms, P4: − 0.07 ms) and Long Evans rats (8 wat P3: − 0.10 ms, P4: − 0.06 ms), while the latencies in Sprague Dawley rats remained stable (8 wat P3: + 0.02 ms, P4: + 0.01 ms). In summary, aside from overall amplitude, these trauma-induced changes in ABR waveforms were small compared to strain-dependent differences found before noise exposure, as emphasized in Fig. 2.

For further quantification of trauma-induced changes at the electrophysiological and behavioral levels and to investigate the possible development of a tinnitus sensation, we compared the pre-trauma startle measurements to the measurements taken 7 and 8 weeks after noise exposure, respectively. Data from Lister Hooded rats were excluded since they exhibited very low ABR amplitudes and lacked inhibition of gap-induced startle responses already before noise exposure (Figs. 1C and 2D). Detailed analysis of trauma-induced changes was only performed on the three remaining strains (SD, WI, LE).

**Fig. 5:**
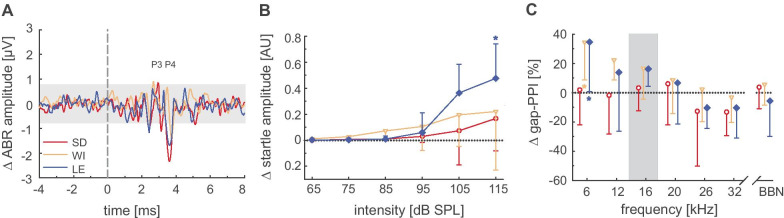
Trauma-induced changes in hearing characteristics. **A** Mean difference in ABR waveforms between pre-trauma values and 8 weeks after noise trauma at 32 kHz/80 dB SPL. The gray area indicates two standard deviations of noise before stimulus onset. Changes above 0 demonstrate a net increase and those below 0 indicate a net decrease in ABR amplitudes. **B** Mean changes in the input/output function of the acoustic startle response 7 weeks after noise trauma. Asterisks indicate significant changes relative to pre-trauma values. **C** Mean changes in gap-prepulse inhibition before and seven weeks after trauma. SD = Sprague Dawley, red open circles, *n* = 8; WI = Wistar, orange open triangles, *n* = 9; LE = Long Evans, blue diamonds *n* = 8

The extent of changes in ABR amplitude was determined by subtracting the individual ABR waveform before noise trauma from the values measured 8 weeks after noise exposure and averaging them for every rat strain (Fig. 5A). Mean amplitudes at 32 kHz show that amplitude values at peaks 3 and 4 changed by more than two standard deviations of the noise floor (shown as the gray area in Fig. 5A), suggesting significant increases in P3 in Sprague Dawley and Wistar rats and decreases in P4 in all three strains, respectively. Regarding the I/O-function of the ASR, we determined, in addition to a clear SPL dependency, significant effects of strain and time on startle amplitude (three-way ANOVA: strain: *F*_2,264_ = 10.8351, *p* < 0.001, time: *F*_1,264_ = 8.65, *p* < 0.001). After noise trauma, startle amplitudes in all three strains increased, and with an increase of 0.48 ± 0.26 (*n* = 8), startle amplitudes were significantly higher for Long Evans rats at 115 dB SPL compared to pre-trauma levels (Fig. 5B). The mean gap-PPI decreased towards higher frequencies 7 weeks after trauma (Fig. 5C) and was significantly affected by the factors strain, frequency, and time (three-way ANOVA: strain: *F*_2,351_ = 58.83, *p* < 0.001, frequency: *F*_7,351_ = 22.71, *p* < 0.001, time: *F*_1,351_ = 8.30, *p* < 0.05). At 6 kHz, only Wistar and Long Evans rats showed significantly increased inhibition values compared to inhibition before trauma. In summary, trauma-induced changes revealed heterogeneous strain dependency at both the behavioral and physiological levels. The strain-dependent pattern was rather complex, and the strongest changes for each of the three different measures (ABR, I/O, gap-PPI) always occurred in a different strain: (i) an increase in ABR P3 amplitude was found in Sprague Dawley and Wistar rats, (ii) ABR P4 amplitude decreased in all strains, (iii) startle amplitude after exposure was increased in Long Evans rats at 115 dB SPL, and (iv) significantly elevated gap-PPI was identified at 6 kHz in Wistar and Long Evans rats. However, all strains investigated revealed a non-significant trend with mean negative gap-PPI values towards higher frequencies, suggesting the development of tinnitus seven weeks after trauma.

### Indications for Tinnitus After Acoustic Trauma

**Fig. 6:**
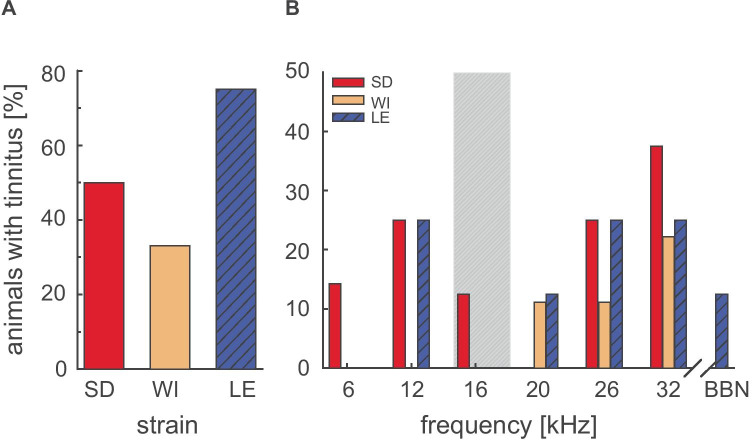
Tinnitus assessment based on the reduction in gap-PPI. **A** General tinnitus occurrence in the three rat strains Sprague Dawley, Wistar, and Long Evans. The indication of tinnitus is measured as the percentage of animals showing reductions in gap-PPI of at least 20% at one or more test frequencies measured 7 weeks after noise trauma. **B** Frequency-specific distribution of tinnitus sensation based on reduction in gap-PPI. The gray bar indicates the frequency range of noise used for acoustic overstimulation. SD = Sprague Dawley, *n* = 8; WI = Wistar, *n* = 9; LE = Long Evans, *n* = 8

A trauma-induced reduction in gap-PPI at a certain test frequency has been suggested to be indicative of tinnitus at the behavioral level (Turner et al. 2006). We quantified such indications of tinnitus at the individual level in all three strains. A decrease in gap-PPI stronger than 20% at a given frequency was taken as an indication of tinnitus (Kiefer et al. 2015). Comparison among strains (Fig. 6A) revealed the highest tinnitus prevalence in Long Evans rats with 75% of the animals being affected. This was followed by Sprague Dawley rats (50%), and the least indication was determined for the Wistar strain (33%). Since we used a narrow noise band centered at around 16 kHz for trauma induction, only parts of the cochlea should be affected. We found that tinnitus indications were most prominent in the high-frequency range around one octave above the center frequencies of the noise exposure at 26 and 32 kHz (Fig. 6B). These high-frequency changes in gap-PPI responses were consistent across all three strains, independent of the degree of hearing loss, as indicated by the change in ABR threshold (see Fig. 3). Hearing thresholds evaluated 1 week after ASR measurements revealed hearing thresholds ≤ 65 dB SPL indicating that the background stimulation of gap-PPI (at 80 dB SPL) was still salient. Furthermore, the frequency-dependent tinnitus indications in Sprague Dawley and Long Evans rats were double-peaked: in addition to the maximum number of affected animals one octave above the trauma frequency, there was an additional maximum immediately below the trauma frequency at approximately 12 kHz. In contrast, Wistar rats were only affected at high frequencies. Therefore, the region one octave above the trauma seems to be most trauma-prone, where gap-PPI-based tinnitus indications are expected first. Taken together, loss of gap-PPI was found in the high-frequency range one octave above the noise trauma frequency in all tested strains. Additionally, loss of gap-PPI occurred in Sprague Dawley and Long Evans rats also at 12 kHz.

### Can Noise-Induced Changes in ABR Amplitude Be Indicative of Tinnitus?

Trauma-induced changes in ABR amplitude as described in the different rat strains (Fig. 4) are usually seen as indicating changes in auditory sensitivity in general. However, recent data indicate specific correlations between ABR growth functions and hearing disorders such as hyperacusis or tinnitus (Möhrle et al. 2019). Following this line of research, we analyzed changes in ABR peak amplitudes at the level of individual animals that might relate to behavioral evidence of tinnitus. Frequencies with the highest tinnitus prevalence (26 and 32 kHz) were chosen for detailed ABR waveform peak analysis. To compare changes in ABR waveforms among strains, the given peak amplitudes were normalized to the respective amplitudes before noise exposure. Therefore, differences among strains in waveform and amplitudes, already present before trauma (Fig. 2), were eliminated.

**Fig. 7:**
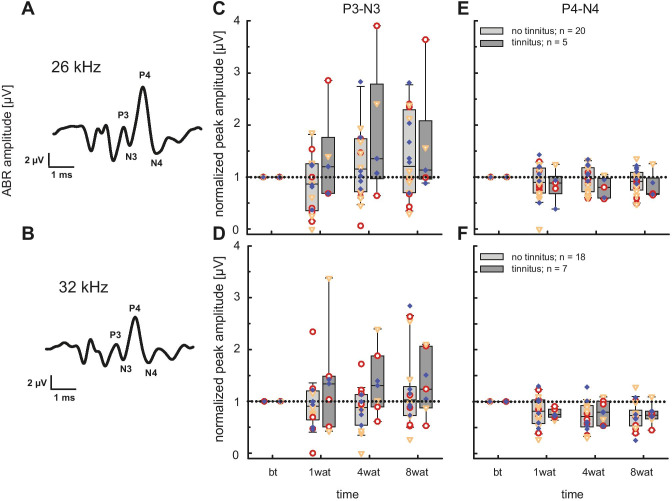
Peak analysis and temporal amplitude change of ABR waveforms. The left column shows the average ABR waveforms of Sprague Dawley rats at **A** 26 kHz and **B** 32 kHz before noise exposure. Peak to trough-amplitudes normalized to pre-trauma (bt = before trauma) of P3-N3 at 26 kHz (**C**) and 32 kHz (**D**). Amplitude data at different time points (wat = weeks after trauma) are plotted separately for animals with tinnitus (light gray) and those without tinnitus (dark gray, stimulation level = 80 dB SPL). Similarly, normalized peak-to-trough amplitudes of P4-N4 at 26 kHz (**E**) and 32 kHz (**F**) over time for tinnitus (light gray) and no-tinnitus (dark gray) animals are displayed. Colored symbols indicate individual animals with their strain affiliations. Red open circles: Sprague Dawley, orange open triangles: Wistar, blue diamonds: Long Evans

For each animal, the changes in peak-to-trough amplitudes of P3-N3 and P4-N4 were calculated relative to pre-trauma amplitudes, at both 26 and 32 kHz (at 80 dB SPL; Fig. 7A, B). Rats were pooled as tinnitus/no-tinnitus animals based on the reduction in gap-PPI (see Fig. 6B). Normalized P3-N3 amplitudes in tinnitus animals were above 1 (Fig. 7C, D), indicating a tendency towards increased amplitude over time after noise exposure for both 26 kHz and (more pronounced) 32 kHz. However, in no-tinnitus animals, P3-N3 amplitudes exhibited a more diverse behavior, especially at 32 kHz, with almost constant amplitudes (Fig. 7D). Conversely, amplitudes of P4-N4 showed smaller variations in the range of approximately 1 across all animals in each group, indicating unchanged amplitude values. In tinnitus animals, a stronger tendency for a decrease rather than an increase was found at both frequencies, more pronounced at 32 kHz (Fig. 7E, F). While absolute change values, as displayed in Fig. 5A, were rather prominent, values normalized and averaged across strains, as shown in Fig. 7E, appear to be comparably small. Most striking was the difference in variation between P3-N3 and P4-N4 amplitudes (Fig. 7C vs. E and D vs. F). However, the small sample size from tinnitus animals prevented deeper statistical analysis and the identification of strain-dependent differences in the change in ABR amplitudes.

## DISCUSSION

Hearing characteristics from four different rat strains underwent comparative analysis to determine and compare the effects of noise exposure. Applying electrophysiological and behavioral measures, we discovered not only strain-dependent features of trauma-induced changes but also remarkable general patterns that appear to be stable across strains and even rodent species. The most consistent characteristic was a frequency-specific pattern of tinnitus prevalence, with the highest rates at approximately one octave above and below the center frequency of acoustic trauma found in Long Evans and Sprague Dawley rats. This resembles what was already described in gerbils, specifically after overstimulation with a broad noise band (Kiefer et al. 2015). Wistar rats, on the other hand, had just one loss of gap-PPI maximum in the higher frequency range. Lister Hooded rats had less sensitive hearing overall and, related, less susceptibility to acoustic overstimulation and were excluded from this analysis because of insufficient previous gap detection performance. The high hearing threshold in Lister Hooded rats was the most prominent strain-specific feature. Interestingly, this occurred in combination with the highest startle amplitude measured among the four strains, which usually indicates sensitive hearing. Furthermore, Lister Hooded rats exhibited minimal inhibition in the gap-PPI paradigm. This might be based on an increased number of high-threshold (auditory nerve) fibers eliciting strong startle responses at high SPL in these rats (Huet et al. 2018). Recruitment might also be involved since the combination of ABR and ASR data suggests a reduced dynamic range in these animals.

The prominent strain-dependent differences in pre-trauma hearing characteristics defined by ABR might result from genetic differences among strains. According to Lindsey and Baker (2006), Sprague Dawley, Wistar, and Long Evans rats are closely related and have a common ancestor, while the origin of Lister Hooded rats remains unknown. This indicates that pigmentation in Long Evans rats has no ancient origin but rather results from crossbreeding. Furthermore, significantly lower pre-trauma hearing thresholds for albino compared to pigmented strains were present, as was found for guinea pigs (Harrison et al. 1984; Conlee et al. 1986).

ABR waveforms showed strain-dependent differences, which were to some extent similar to previous descriptions (Chen and Chen, 1990; Overbeck and Church, 1992). Strain-dependent differences in hearing sensitivity could largely explain the variations in ABR waveforms at 80 dB SPL. The low ABR amplitudes found in Lister Hooded rats in combination with high hearing thresholds might indicate that the activity of neurons along the auditory pathway in this strain is not as tightly synchronized as in the others.

Differences in startle amplitudes among strains (see I/O-functions in Fig. 1B) followed a pattern markedly different from the strain-dependent pattern of ABR thresholds. Lister Hooded rats, with the least sensitive hearing, showed the highest startle amplitudes, while Sprague Dawley and Long Evans rats, with the most sensitive hearing, had the lowest startle amplitudes. Different from maximal startle amplitudes, almost identical ASR thresholds were found in all strains tested, despite their rather different ABR thresholds. This matches the assumption that the ASR threshold is determined by more peripheral mechanisms, while ABR thresholds, usually based on the evaluation of a late, large peak component, e.g., P4 (see Fig. 2), evaluate the sensitivity of the upper brainstem (presumably the inferior colliculus).

Gap-PPI measurements revealed strain-dependent differences that could not be predicted from the patterns of hearing sensitivity and startle responsiveness (ABR or ASR amplitude). The inhibition in Sprague Dawley rats (78%) was highest followed by Long Evans (58%) and Wistar rats (44%). Finally, Lister Hooded rats showed very low (sometimes negative) levels of gap-PPI, which is related to their insensitive hearing, as indicated by ABR thresholds (see Fig. 1A). Previous studies on different gap-PPI levels in individual rat strains support this observation: the inhibition level in Wistar and Long Evans rats was comparable to findings by Liu and Chen (2015) and Mao et al. (2012), respectively. No literature data on gap-PPI are available for Lister Hooded rats. This strain had a mean inhibition of − 13% but exhibited a frequency-dependent pattern of inhibition that paralleled the pattern found in the other strains. This points towards a mechanism of gap facilitation in the low-frequency range, as it was induced by salicylate in Sprague Dawley rats (Sun et al. 2014). In additional experiments on two Lister Hooded rats, we found a stronger gap-PPI (57–69%) for shorter gap lengths (100 ms instead of 500 ms, data not shown). This indicates that Lister Hooded rats can perceive background noise and that gap-in-noise can be used as a prepulse. However, these data suggest a temporal processing deficit in Lister Hooded rats compared to other strains.

Noise-exposure-induced changes in peripheral hearing revealed further differences among the four rat strains. The induced damage to the auditory system resulted in significant ABR threshold elevations in all strains. All normal-hearing rat strains exhibited the highest threshold shifts of approximately 50 dB at 26 and 32 kHz stimulus frequencies directly after trauma. Conversely, click responses remained unaffected by trauma in all rat strains. The lower limit of the frequency range being affected by the trauma (as indicated by threshold shift directly after trauma) varied in correlation with pigmentation of the animals. Albino strains showed a threshold shift at and above 16 kHz, while pigmented strains already experienced a shift at and above 12 kHz. Possibly, a general difference in the susceptibility to the traumatizing noise (around 16 kHz) might explain these differences.

The time course of recovery of ABR thresholds after trauma was strain-dependent: 1 week after trauma, the hearing thresholds of Lister Hooded and Long Evans rats had returned to pre-trauma levels, while significant threshold elevations in Sprague Dawley and Wistar rats were still observed at 32 kHz. This elevation persisted in Sprague Dawley rats, while Wistar rats had recovered ABR thresholds at all frequencies tested by the end of the measurements. These findings indicate a permanent threshold shift (PTS) in Sprague Dawley rats at a frequency one octave above the center frequency of the traumatizing noise band, while a temporary threshold shift (TTS) in Wistar rats was found at this frequency. TTS seems to be common in Wistar rats and has been described several times (Zheng et al. 2011, 2014; Rüttiger et al. 2013; Singer et al. 2013; Bing et al. 2015). Although a complete recovery of thresholds was observed one week after trauma in Long Evans rats, a significant threshold elevation at 32 kHz reappeared starting 4 weeks after trauma and persisted further. This finding might indicate that a PTS is occurring in this strain but with a later onset than in albino strains. It is possible that these pigmented animals have a short-term compensation mechanism that eventually breaks down, and long-term damage following overstimulation emerges.

The test of tinnitus prevalence based on a loss of gap-PPI revealed strain- and frequency-dependent differences: 33% of Wistar, 50% of Sprague Dawley, and 75% of Long Evans rats demonstrated behavioral correlates of tinnitus. Overall, the tinnitus rates observed for Long Evans and Wistar rats are in line with previous results (Zhang et al. 2011, Rüttiger et al. 2013). Regarding Sprague Dawley rats, in the present study, 50% of Sprague Dawley rats showed behavioral evidence of tinnitus, while a broad range of tinnitus rates, from 37 to 86%, has been reported in the literature (Kraus et al. 2011; Lobarinas et al. 2015; Pace et al. 2016). There is an ongoing debate of co-detecting hyperacusis and hearing loss by measuring gap-PPI (Chen et al. 2013; Hayes et al. 2014; Hickox and Liberman 2014; Salloum et al. 2016). Thus, we made sure to control for those: ABR thresholds of all animals tested for tinnitus were ≥ 15 dB below the intensity of the background noise, making the background stimuli salient. Hearing loss alone did not predict the occurrence of tinnitus as many animals showed loss of gap-PPI at 26 kHz as well. Furthermore, no signs of hyperacusis with increased reactivity to background stimulation according to previous publications were detected (Chen et al. 2013; Salloum et al. 2016). Some studies classify increased ASR amplitudes as hyperacusis (Sun et al. 2012; Hickox and Liberman 2014; Salloum et al. 2014). However, changes in ASR amplitudes occur in response to sounds with high intensities, whereas an increased loudness perception at moderate levels defines hyperacusis, making this aspect of ASR inappropriate for diagnosing hyperacusis (Hayes et al. 2014; Knudson and Melcher 2016).

Changes in ABR waveform components partly reflect the observed threshold changes: directly after trauma, the waveforms in all strains were not discernible from noise at a stimulus intensity of 80 dB SPL. While the amplitude of P4 is slightly decreased, earlier peaks show an increased amplitude in comparison to pre-trauma values. This could indicate compensation taking place along the auditory pathway, reflecting more synchronized activity. The increase in normalized P3 amplitude, especially in tinnitus animals, also points to a compensation mechanism in the peripheral part of the auditory pathway since P4 amplitudes do not change after trauma, as previously shown in mice (Schrode et al. 2018). Nonetheless, this was most pronounced at 32 kHz, while it disappeared eight weeks after trauma at 26 kHz. Thus, it seems possible that hearing loss promotes a long-lasting compensation mechanism that eventually leads to a tinnitus sensation. However, we found a weak relationship between ABR waveform and tinnitus. P4 latencies at 32 kHz decreased in half of the tinnitus animals. Further, a recent study on humans suggests a reduced latency of the IC peak to correspond to a co-occurrence of tinnitus and hyperacusis (Refat et al. 2021). Thus, it might be possible that a subset of tinnitus animals also experienced hyperacusis.

## CONCLUSION

We found crucial strain-dependent differences in rats even before they were exposed to noise trauma. Furthermore, susceptibility to noise trauma and the occurrence of gap-PPI reduction were clearly strain-dependent. Despite clear differences in hearing sensitivity among all tested strains, we observed a commonality: Sprague Dawley and Long Evans rats exhibited most sensitive pre-trauma hearing alongside highest responses in gap-PPI, had a PTS after trauma, and revealed the highest tinnitus rates. Overall, this led to astonishingly different characterizations of the four rat strains investigated. Conversely, one startle-derived measure commonly used for tinnitus characterization provided a consistent picture: the gap-PPI-based measure for frequency-dependent tinnitus prevalence was consistent across three out of four strains. This reproducible tinnitus characterization is a promising starting point for studies on the mechanisms underlying tinnitus and related trauma-induced hearing problems.

## Data Availability

Data can be shared with the authors upon request.

## Abbreviations

ABR: Auditory brainstem response
ASR: Acoustic startle response
BBN: Broadband noise
BW: Body weight
I/O: Input/Output
LE: Long Evans
LH: Lister Hooded
PPI: Prepulse inhibition
PTS: Permanent threshold shift
SD: Sprague Dawley
TTS: Temporary threshold shift
wat: Weeks after trauma
WI: Wistar

## Code Availability

Code can be shared with the authors upon request.

All procedures were in accordance with ethical standards for the care and use of animals in research, the German Animal Welfare Act, and were approved by the Regierungspraesidium Darmstadt (FU/Anz. 1002).
